# The prevalence and correlates of peripartum depression in different stages of pregnancy during COVID-19 pandemic in China

**DOI:** 10.1186/s12884-022-04428-1

**Published:** 2022-02-11

**Authors:** Manji Hu, Yongjie Zhou, Mei Xue, Yali Ren, Shen Li, Ruoxi Wang, Ling Qi, Lingyun Zeng, Zhengkui Liu, Wei Qian, Jiezhi Yang, Xin Zhou, Lijuan Chen, Xiangyang Zhang

**Affiliations:** 1 Shanghai Pudong New Area Mental Health Centre, Shanghai, China; 2 Department of Psychiatric Rehabilitation, Shenzhen Kangning Hospital, Shenzhen, Guangdong China; 3 Qingdao Mental Health Center, Qingdao University, Qingdao, China; 4 Department of Medical Affairs, Liyuan Hospital of Tongji Medical College, Huazhong University of Science and Technology, 39 Yanhu Road, Wuchang District, Wuhan, 430077 Hubei China; 5 Department of Psychiatry, College of Basic Medical Sciences, Tianjin Medical University, Tianjin, China; 6 School of Medicine and Health Management, Tongji Medical College, Huazhong University of Science and Technology, Wuhan, China; 7 School of Health Science and Nursing, Wuhan Polytechnic University, Wuhan, China; 8 CAS Key Laboratory of Mental Health, Institute of Psychology, Chinese Academy of Sciences, Beijing, China; 9 Shenzhen Health Development Research Center, Shenzhen, China; 10 Research Center for Psychological and Health Sciences, China University of Geosciences, Wuhan, China; 11 School of Literature, Journalism & Communication, South-Central University for Nationalities, Wuhan, China

**Keywords:** Peripartum depression, Pregnancy, Pregnant women, Puerperant, COVID-19

## Abstract

**Background:**

Peripartum depression in and after pregnancy are common, reported by 11.9% of women worldwide, and the proportion was even higher during the outbreak of coronavirus disease 2019 (COVID-19). We aimed to investigate the prevalence and risk factors of peripartum depression under the influence of COVID-19 in China.

**Methods:**

Using a cross-sectional design, 2026 pregnant and postpartum women residing in Beijing, Wuhan, and Lanzhou of China were recruited from February 28 to April 9, 2020. The Patient Health Questionnaire-9 was used to assess their depressive symptoms. The women were divided into four subgroups based on pregnancy stage, and a binary logistic regression analysis was conducted on each subgroup.

**Results:**

Under the influence of COVID-19, the prevalence rate of peripartum depression among Chinese women was 9.7%. It was 13.6, 10.8, 7.9 and 7.3% in the first, second, third trimester and puerperium, respectively. Regression analysis showed that the influence of current pregnancy status on movement (Mild vs. No, aORs were 3.89, *P* < 0.001, 2.92, *P* = 0.003, 1.58, *P* = 0.150 in the three trimesters, respectively; Severe vs. No, aORs were 13.00, 20.45, 5.38 in the three trimesters, respectively, all *P* < 0.05), and worries and fears about childbirth (aORs were 2.46, 2.96, 2.50 in the three trimesters, respectively, all *P* < 0.05) were associated with depression throughout pregnancy.

**Conclusions:**

The prevalence rate of peripartum depression during the COVID-19 outbreak in China was not higher than usual. The influence of current pregnancy status on movement, as well as worries and fears about childbirth were independent risk factors for peripartum depression throughout pregnancy during COVID-19. The stage of pregnancy should be considered when implementing interventions.

## Background

More and more attention has been paid to postpartum depression. However, insufficient attention has been paid to peripartum depression, which is defined as a major depressive episode during pregnancy and/or within 4 weeks after delivery from the Diagnostic and Statistical Manual of Mental Disorders-5 [1]. By definition, the duration of peripartum depression is wider than that of postpartum depression. Peripartum depression not only seriously affects the physical and mental health of pregnant women and puerperants, but also has many negative effects on the family and fetus, and even late infancy and childhood [2]; for example, peripartum depression can incapacitate mothers [3], increase the risk of preterm birth, alter the neurodevelopment of the fetus [4], and make school-age children more prone to aggressive behavior and learning difficulties [3]. Risk factors for peripartum depression include domestic violence [3], physical dissatisfaction [5], low social support [6], history of depression, stressful life events, etc.

According to a previous review, 11.9% of women worldwide suffer from peripartum depression [7]. It has been reported that before and after the COVID-19 epidemic was announced, the prevalence rate of depressive symptoms among Chinese women in the third trimester of pregnancy was as high as 26.0 and 29.6%, respectively [8], suggesting that the rate of depression increased by 3.6% after COVID-19 was identified. A sample survey study in Turkey showed that during the COVID-19 pandemic, 35.4% of pregnant women had a score more than 13 on the Edinburgh Postnatal Depression Scale (EPDS) [9]. Social and psychological stressors increased sharply during the coronavirus disease 2019 (COVID-19) pandemic [10].

However, studies on peripartum depression in China are not representative. At present, the domestic literature is mostly concentrated in a single city or a single pregnancy stage, and the sample sizes are small. In addition, the COVID-19 pandemic has strong infectivity, great influence and wide spread [11]. The purpose of this study was to understand the impact of COVID-19 pandemic on maternal depression in China, and to explore the related factors, in order to make up for the lack of research on peripartum depression in China, and to develop peripartum depression interventions on this basis.

## Methods

### Design and setting

We assumed that the prevalence rate of peripartum depression during the COVID-19 pandemic outbreak was higher than usual. A cross-sectional design was adopted and a structured self-assessment questionnaire through the online questionnaire platform “Survey Star” (Changsha Ranxing Information Technology Co., Ltd.) was established. People filled out the electronic version of the questionnaire in Chinese through their mobile phones, and the platform collected the questionnaire information to us. The contents of the questionnaire included demographic information (20 questions) and depressive symptoms (9 questions), with a total of 29 questions. Using the method of multi-stage sampling technique, 2236 women were recruited from February 28 to April 9, 2020. The first stage was intentional sampling, in which Beijing, Lanzhou and Wuhan were selected according to the severity of the pandemic and economic development. The reasons for choosing these three cities were as follows. From the perspective of COVID-19 pandemic, Wuhan was the most serious area with the largest number of cumulative confirmed cases, followed by Beijing and Lanzhou. The order of economic development from most to least is Beijing, Wuhan, and Lanzhou. In the second stage, convenience sampling was adopted. The quick response (QR) code of the questionnaire was sent to the investigators in the three cities, and then they sent the QR code to the staff of medical institutions at all levels that were qualified for maternal examination. According to China’s maternal health policy, pregnant and postpartum women are required to go to the hospital regularly for antepartum or postpartum checkups, including, but not limited to weight, blood pressure, fetal heart rate, uterine height, etc. These women who came to the hospital for examination were the subjects of our survey. They were asked to voluntarily scan the QR code on their mobile phones to fill out the questionnaire. These women were divided into four subgroups based on pregnancy stages [12, 13]: the first trimester of pregnancy (< 14 weeks of gestation), the second trimester of pregnancy (14-28 weeks of gestation), the third trimester of pregnancy (≥29 weeks of gestation), puerperal period (within 6 weeks after delivery).

**Fig. 1:**
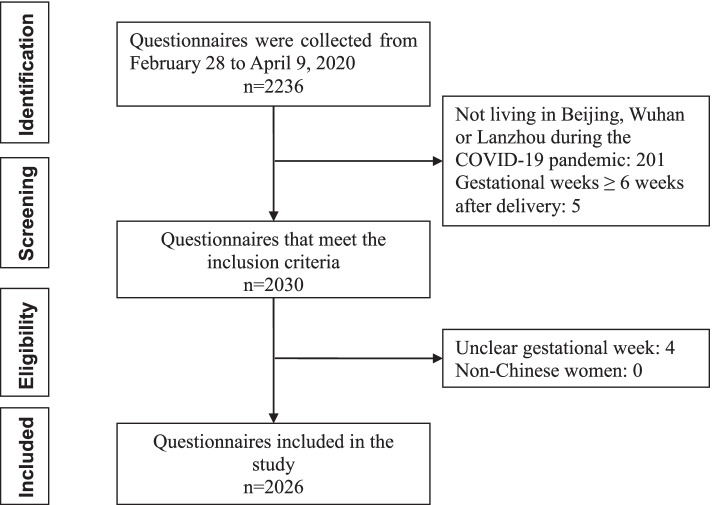
The flowchart of all questionnaires eligible in the study

The inclusion criteria were: (1) women from the beginning of pregnancy to 6 weeks after delivery (considering the definition of the puerperal period, the time range of peripartum depression was extended to 6 weeks after delivery); and (2) living in Beijing, Wuhan or Lanzhou during the COVID-19 pandemic. The exclusion criteria were: (1) unclear gestational week; and (2) non-Chinese women. Finally, 2026 questionnaires were included in the study. The flowchart of all questionnaires eligible is shown in Fig. 1.

The ethical approval in line with the Declaration of Helsinki was granted by the Ethics Committee of the Institute of Psychology, Chinese Academy of Sciences. All participants signed the informed consent form before the start of the study.

### Demographic information

Three aspects of demographic information were collected, namely, basic information, pregnancy-related information and COVID-19-related information. The basic data included resident city, age, height, weight, marital status, education level, family income level, medication, physical diseases history, daily smoking, and daily alcohol use. These sociodemographic variables were selected referring to previous studies [5, 8, 14, 15] and points of interest. Information related to pregnancy included gestational weeks, parity, severity level of vomiting during pregnancy, significant uterine contractions caused by anxiety, the influence of current pregnancy status on movement (act of moving the body or part of the body), worries and fears about childbirth (fear of pain during childbirth and the danger of childbirth), care of daily life by others, living status with parents-in-law, and living status with parents. Information related to COVID-19 included economic losses caused by COVID-19, COVID-19 infection status of pregnant women and their relatives and friends.

### Depressive symptoms

The Chinese version of the Patient Health Questionnaire-9 (PHQ-9) was used to evaluate depressive symptoms. This scale is open and free. It is widely used to measure the level of depression in the population in China [16, 17]. However, many studies chose EPDS as an evaluation tool. In fact, both PHQ-9 and EPDS are reliable and effective in assessing antepartum depression. Many scholars have proved that there was no significant difference between PHQ-9 and EPDS in detecting Major Depressive Disorder diagnosed by clinicians [18–20]. The PHQ-9 mainly measures somatic symptoms, while EPDS mainly assessed symptoms of depression and anxiety in early pregnancy [21]. In addition, this manuscript studies the symptom of depression, not depression with anxiety symptoms. Anxiety disorder has a special rating scale (GAD-7) in our entire research project. Therefore, PHQ-9 was chosen to evaluate depression symptoms during pregnancy and puerperium. Participants were asked to assess the frequency of depressive symptoms they experienced within the past 2 weeks. It is an ordinal scale, from 0 (not at all) to 3 (almost every day) for a total of 4 levels. It was developed according to the DSM-4 criteria for depressive disorder. The total score of all 9 items reflects the severity of depression, ranging from 0 to 27. According to previous studies [22, 23], a score of ≥10 in diagnosing major depressive disorder has good sensitivity and specificity, and it has been proved working well in pregnant women. Thus, a score of 10 or above indicates major depression [24], and was divided into “depression” group, while a score of less than 10 points was divided into “non-depression” group. It is worth mentioning that the PHQ-9 cutoff score of 10 has not been recommended for pregnant women specifically, which is the recommendation for the general population.

### Statistical analyses

The categorical data were expressed as proportions, and the differences between rates were tested by chi-square or Fisher exact tests, if appropriate. The rest of the data were continuous variables. After the Kolmogorov-Smirnov test, we used the median and interquartile range (IQR) to describe those data that were not normally distributed. And the rank sum test was used for the comparison between groups. Internal reliability of PHQ-9 results was assessed by Cronbach’s alpha. Because the dependent variable did not match the normal distribution and had the problem of collinearity, the binary logistic regression (step forward likelihood ratio approach into analysis) was used to carry out multi-factor analysis. Adjusted odds ratio (aOR) values and 95% confidence intervals (CI) were calculated. These statistical analyses were performed using IBM SPSS statistics version 21.0, and the results were considered to be statistically significant if the two-tailed *p* < 0.05.

## Results

### Sample characteristics

**Fig. 2:**
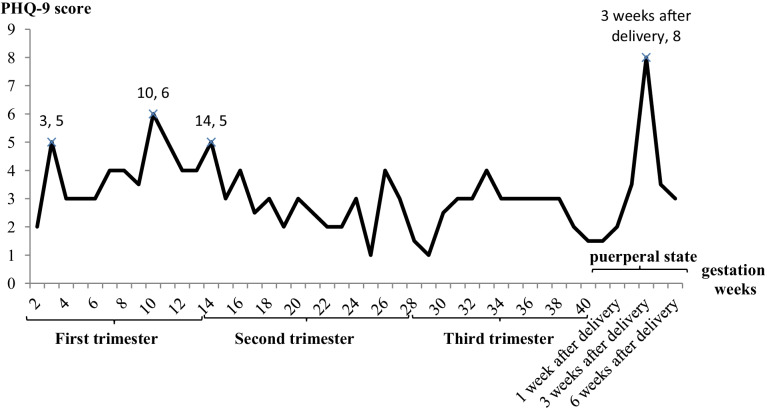
The median PHQ-9 score of women in different stages of pregnancy. PHQ-9, the Patient Health Questionnaire-9

The median and IQR of PHQ-9 scores of all participants were 3.0 and 1.0-6.0, respectively. The prevalence rate of peripartum depressive symptoms (PHQ-9 score ≥ 10) was 9.7% (197/2026), and PHQ-9 demonstrated adequate internal consisitency reliability with a Cronbach’s coefficient alpha of 0.854 for this sample. The prevalence rate of peripartum depressive symptoms was 13.6% in the first trimester (56/411), 10.8% in the second trimester (53/491), 7.9% in the third trimester (82/1042) and 7.3% in the puerperium (6/82). Basic descriptions of other characteristics are summarized in Table 1. The median PHQ-9 scores of women in different weeks of pregnancy are shown in Fig. 2. As shown in Fig. 2, depressive symptoms were most serious in the 3rd week of postpartum, followed by the 10th week of pregnancy, and then followed by the 3rd and 14th week of pregnancy.

**Table 1:** Sample characteristics of participants

Characteristics	n (%)	Median (IQR)
Resident city		
Beijing	827(40.8)	
Lanzhou	434(21.4)	
Wuhan	765(37.8)	
Age (year)		30.0(28.0-33.0)
Height (cm)		162.0(158.1-165.0)
Weight (kg)		65.0(57.0-72.0)
Marital (divorced/unmarried)	36(1.7)	
Education level		
Junior high school or below	68(3.3)	
Senior high school / technical secondary school	239(11.8)	
Junior college	587(29.0)	
Bachelor	900(44.4)	
Postgraduate	232(11.5)	
Family income (yearly, Yuan)		
80 thousand or below	594(29.3)	
80 thousand to 0.3 million	1168(57.7)	
More than 0.3 million	264(13.0)	
Economic losses caused by COVID-19 (Thousand Yuan)		2.0(0.0-5.0) [73]
Primipara	1401(69.2)	
History of physical diseases	395(19.5)	
History of mental illness	14(0.7)	
Taking medication (any medication)	189(9.3)	
Daily smoking	6(0.3)	
Daily alcohol use	39(1.9)	
Vomiting during pregnancy		
None	544(26.9)	
Mild (self-remission)	1330(65.6)	
Severe (ask for treatment)	152(7.5)	
Significant uterine contractions caused by anxiety	500(24.7)	
The influence of current pregnancy status on movement		
No	845(41.7)	
Mild	1108(54.7)	
Severe	73(3.6)	
Have worries and fears about childbirth	707(34.9)	
Requiring other people to help with daily tasks most of the time	1508(74.4)	
Living with parents-in-law	549(27.1)	
Living with parents	421(20.8)	
COVID-19 infection status of pregnant women and their relatives and friends	17(0.8)	

Numbers in brackets refer to number of missing values

*IQR* Interquartile range

### Comparisons between depressive symptoms group and non-depressive symptoms group in different stages of pregnancy

The comparisons of participants’ characteristics in different stages of pregnancy between the depressive symptoms group (PHQ-9 score ≥ 10) and the non-depressive symptoms group (PHQ-9 score < 10) are shown in Tables 2, 3, 4 and 5. In the first trimester, there were statistically significant differences between the two groups in the following variables: resident city, marital status, family income level, history of mental illness, severity level of vomiting during pregnancy, the influence of current pregnancy status on movement and worries and fears about childbirth. In the second trimester, there were statistically significant differences between the two groups in these variables: resident city, age, family income level, economic losses caused by COVID-19, the influence of current pregnancy status on movement, worries and fears about childbirth, care for daily life by others and living status with parents-in-law. In the third trimester, there were statistically significant differences between the two groups in resident city, economic losses caused by COVID-19, significant uterine contractions caused by anxiety, the influence of current pregnancy status on movement and worries and fears about childbirth. During puerperium, there were statistically significant differences between the two groups in age, weight, body mass index (BMI), history of physical diseases and the influence of current pregnancy status on movement.

**Table 2:** Comparison between the “depression” group and the “non-depression” group in the first trimester of pregnancy

Characteristics	Non-depressive symptoms^**c**^ (***n*** = 355)	Depressive symptoms^**c**^ (***n*** = 56)	***P***
Resident city			< 0.001
Beijing	204(57.5%)	15(26.8%)	
Lanzhou	121(34.0%)	35(62.5%)	
Wuhan	30(8.5%)	6(10.7%)	
Age (year)^d^	30.0(28.0-32.0)	30.0(26.2-32.0)	0.223
Height (cm)^d^	163.0(159.0-166.0)	160.5(159.2-166.0)	0.419
Weight (kg) ^d^	57.0(52.0-63.5)	55.0(51.7-61.9)	0.230
Marital status (divorced/unmarried)	9(2.5%)	5(8.9%)	0.040^b^
Education level			0.151^a^
Junior high school or below	7(2.0%)	3(5.4%)	
Senior high school/technical secondary school	25(7.0%)	5(9.0%)	
Junior college	93(26.2%)	18(32.1%)	
Bachelor	171(48.2%)	26(46.4%)	
Postgraduate	59(16.6%)	4(7.1%)	
Family income (yearly, Yuan)			0.008
80 thousand or below	98(27.6%)	27(48.2%)	
80 thousand to 0.3 million	195(54.9%)	22(39.3%)	
More than 0.3 million	62(17.5%)	7(12.5%)	
Economic losses caused by COVID-19 (Thousand Yuan) ^d^	2.0(0.0-5.0)[16]	2.0(0.2-5.0)[3]	0.642
Primipara	266(74.9%)	44(78.6%)	0.556
History of physical diseases	49(13.8%)	10(17.9%)	0.421
History of mental illness	1(0.3%)	2(3.6%)	0.050^a^
Taking medication	36(10.1%)	6(10.7%)	0.895
Daily smoking	2(0.6%)	0(0.0%)	1.000^a^
Daily alcohol use	14(3.9%)	1 (1.8%)	0.677^b^
Vomiting during pregnancy			< 0.001
None	97(27.3%)	6(10.7%)	
Mild (self-remission)	236(66.5%)	37(66.1%)	
Severe (ask for treatment)	22(6.2%)	13(23.2%)	
Significant uterine contractions caused by anxiety	9(2.5%)	3(5.4%)	0.460^b^
The influence of current pregnancy status on movement			< 0.001
No	219(61.7%)	17(30.4%)	
Mild	133(37.5%)	34(60.7%)	
Severe	3(0.8%)	5(8.9%)	
Have worries and fears about childbirth	95(26.8%)	24(42.9%)	0.014
Requiring other people to help with daily tasks most of the time	238(67.0%)	33(58.9%)	0.234
Living with parents-in-law	54(15.2%)	7(12.5%)	0.596
Living with parents	52(14.6%)	9(16.1%)	0.781
COVID-19 infection status of pregnant women and their relatives and friends	2(0.6%)	0(0.0%)	1.000^a^

Numbers in brackets refer to number of missing values

*COVID-19* 2019 coronavirus disease

^a^Fisher exact test

^b^Continuous correction of chi-square test

^c^The value are given as the number of participant or median with the percentage or interquartile range in parentheses, respectively

^d^Because these data were not normally distributed, the rank sum test was used for the comparison between groups

**Table 3:** Comparison between the “depression” group and the “non-depression” group in the second trimester of pregnancy

Characteristics	Non-depressive symptoms^**c**^ (***n*** = 438)	Depressive symptoms^**c**^ (***n*** = 53)	***P***
Resident city			0.031
Beijing	180(41.1%)	13(24.5%)	
Lanzhou	111(25.3%)	21(39.6%)	
Wuhan	147(33.6%)	19(35.8%)	
Age (year)^d^	30.0(28.0-33.0)	29.0(25.0-32.0)	0.026
Height (cm)^d^	162.0(158.0-165.0)	160.0(157.7-164.0)	0.101
Weight (kg) ^d^	60.0(55.0-67.0)	59.0(53.0-65.5)	0.425
Marital status (divorced/unmarried)	13(3.0%)	4(7.5%)	0.185^b^
Education level			0.057^a^
Junior high school or below	17(3.9%)	4(7.5%)	
Senior high school/technical secondary school	47(10.7%)	7(13.2%)	
Junior college	119(27.2%)	22(41.5%)	
Bachelor	202(46.1%)	16(30.2%)	
Postgraduate	53(12.1%)	4(7.5%)	
Family income (yearly, Yuan)			< 0.001
80 thousand or below	126(28.8%)	30(56.6%)	
80 thousand to 0.3 million	248(56.6%)	18(34.0%)	
More than 0.3 million	64(14.6%)	5(9.4%)	
Economic losses caused by COVID-19 (Thousand Yuan) ^d^	2.0(0.0-5.0)[14]	3.7(2.0-5.0)[1]	0.009
Primipara	317(72.4%)	41(77.4%)	0.441
History of physical diseases	56(12.8%)	6(11.3%)	0.762
History of mental illness	4(0.9%)	0(0.0%)	1.000^a^
Taking medication	35(8.0%)	8(15.1%)	0.141 ^b^
Daily smoking	1(0.2%)	0(0.0%)	1.000^a^
Daily alcohol use	4(0.9%)	1 (1.9%)	0.437^a^
Vomiting during pregnancy			0.533
None	113(25.8%)	17(32.1%)	
Mild (self-remission)	298(68.0%)	32(60.4%)	
Severe (ask for treatment)	27(6.2%)	4(7.5%)	
Significant uterine contractions caused by anxiety	38(8.7%)	9(17.0%)	0.078^a^
The influence of current pregnancy status on movement			< 0.001
No	229(52.3%)	13(24.5%)	
Mild	206(47.0%)	37(69.8%)	
Severe	3(0.7%)	3(5.7%)	
Have worries and fears about childbirth	118(26.9%)	29(54.7%)	< 0.001
Requiring other people to help with daily tasks most of the time	310(70.8%)	26(49.1%)	0.001
Living with parents-in-law	80(18.3%)	16(30.2%)	0.039
Living with parents	90(20.5%)	7(13.2%)	0.205
COVID-19 infection status of pregnant women and their relatives and friends	6(1.4%)	0(0.00%)	1.000^a^

Numbers in brackets refer to number of missing values

*COVID-19* 2019 coronavirus disease

^a^Fisher exact test

^b^Continuous correction of chi-square test

^c^The value are given as the number of participant or median with the percentage or interquartile range in parentheses, respectively

^d^Because these data were not normally distributed, the rank sum test was used for the comparison between groups

**Table 4:** Comparison between the “depression” group and the “non-depression” group in the third trimester of pregnancy

Characteristics	Non-depressive symptoms^**c**^ (***n*** = 960)	Depressive symptoms^**c**^ (***n*** = 82)	***P***
Resident city			0.003
Beijing	391(40.7%)	18(22.0%)	
Lanzhou	133(13.9%)	13(15.9%)	
Wuhan	436(45.4%)	51(62.1%)	
Age (year)^d^	30.0(28.0-32.0)	31.0(28.0-33.0)	0.208
Height (cm)^d^	162.0(158.5-165.0)	162.0(158.0-165.0)	0.738
Weight (kg) ^d^	69.5(63.0-75.0)	69.9(63.9-77.7)	0.414
Marital status (divorced/unmarried)	3(0.3%)	2(2.4%)	0.052^a^
Education level			0.539^a^
Junior high school or below	29(3.0%)	3(3.7%)	
Senior high school/technical secondary school	129(13.5%)	12(14.6%)	
Junior college	280(29.2%)	30(36.6%)	
Bachelor	419(43.6%)	31(37.8%)	
Postgraduate	103(10.7%)	6(7.3%)	
Family income (yearly, Yuan)			0.178
80 thousand or below	262(27.3%)	20(24.4%)	
80 thousand to 0.3 million	583(60.7%)	57(69.5%)	
More than 0.3 million	115(12.0%)	5(6.1%)	
Economic losses caused by COVID-19 (Thousand Yuan) ^d^	2.0(0.5-5.0)[31]	4.0(2.0-6.5)[4]	0.003
Primipara	658(68.5%)	59(72.0%)	0.522
History of physical diseases	229(23.9%)	23(28.0%)	0.395
History of mental illness	6(0.6%)	1(1.2%)	0.438^a^
Taking medication	86(9.0%)	12(14.6%)	0.091
Daily smoking	3(0.3%)	0(0.0%)	1.000^a^
Daily alcohol use	14(1.5%)	2(2.4%)	0.822^b^
Vomiting during pregnancy			0.069^a^
None	275(28.6%)	18(22.0%)	
Mild (self-remission)	620(64.6%)	53(64.6%)	
Severe (ask for treatment)	65(6.8%)	11(13.4%)	
Significant uterine contractions caused by anxiety	375(39.1%)	46(56.1%)	0.003
The influence of current pregnancy status on movement			< 0.001
No	333(34.7%)	15(18.3%)	
Mild	591(61.6%)	54(65.9%)	
Severe	36(3.8%)	13(15.9%)	
Have worries and fears about childbirth	354(36.9%)	53(64.6%)	< 0.001
Requiring other people to help with daily tasks most of the time	772(80.4%)	60(73.2%)	0.116
Living with parents-in-law	333(34.7%)	27(32.9%)	0.748
Living with parents	221(23.0%)	15(18.3%)	0.326
COVID-19 infection status of pregnant women and their relatives and friends	5(0.5%)	1(1.2%)	0.389^a^

Numbers in brackets refer to number of missing values

*COVID-19* 2019 coronavirus disease

^a^Fisher exact test

^b^Continuous correction of chi-square test

^c^The value are given as the number of participant or median with the percentage or interquartile range in parentheses, respectively

^d^Because these data were not normally distributed, the rank sum test was used for the comparison between groups

**Table 5:** Comparison between the “depression” group and the “non-depression” group in puerperal period

Characteristics	Non-depressive symptoms^**c**^ (***n*** = 76)	Depressive symptoms^**c**^ (***n*** = 6)	***P***
Resident city		1.000^b^	1.000^a^
Beijing	6(7.9%)	0(0.0%)	
Lanzhou	0(0.0%)	0(0.0%)	
Wuhan	70(92.1%)	6(100%)	
Age (year)^d^	30.0(28.0-33.0)	34.5(31.2-36.7)	0.029
Height (cm)^d^	162.0(159.0-164.7)	159.5(154.5-162.7)	0.171
Weight (kg) ^d^	64.9(60.0-70.0)	81.0(78.7-85.5)	0.001
BMI (kg/m^2^) ^d^	25.3(22.6-27.3)	32.2(31.0-33.6)	< 0.001
Marital status (divorced/unmarried)	0(0.0%)	0(0.0%)	–
Education level			0.415^a^
Junior high school or below	4(5.3%)	1(16.7%)	
Senior high school/technical secondary school	12(15.8%)	2(33.3%)	
Junior college	24(31.6%)	1(16.7%)	
Bachelor	33(43.4%)	2(33.3%)	
Postgraduate	3(3.9%)	0(0.0%)	
Family income (yearly, Yuan)			0.802^a^
80 thousand or below	28(36.8%)	3(50%)	
80 thousand to 0.3 million	42(55.3%)	3(50%)	
More than 0.3 million	6(7.9%)	0(0.0%)	
Economic losses caused by COVID-19 (Thousand Yuan) ^d^	3.0(2.0-9.5)[4]	4.0(1.7-6.5)	0.977
Primipara	15(19.7%)	1(16.7%)	1.000^a^
History of physical diseases	17(22.4%)	5(83.3%)	0.005^a^
History of mental illness	0(0.0%)	0(0.0%)	
Taking medication	0(0.0%)	0(0.0%)	1.000^a^
Daily smoking	3(3.9%)	0(0.0%)	1.000^a^
Daily alcohol use	7(9.2%)	0(0.0%)	1.000^a^
Vomiting during pregnancy			1.000^a^
None	17(22.4%)	1(16.7%)	
Mild (self-remission)	50(65.8%)	4(66.6%)	
Severe (ask for treatment)	9(11.8%)	1(16.7%)	
Significant uterine contractions caused by anxiety	18(23.7%)	2(33.3%)	0.630^a^
The influence of current pregnancy status on movement			0.028^a^
No	19(25.0%)	0(0.0%)	
Mild	50(65.8%)	3(50.0%)	
Severe	7(9.2%)	3(50.0%)	
Have worries and fears about childbirth	31(40.8%)	3(50.0%)	0.688^a^
Requiring other people to help with daily tasks most of the time	65(85.5%)	4(66.7%)	0.524^a^
Living with parents-in-law	30(39.5%)	2(33.3%)	1.000^a^
Living with parents	23(30.3%)	4(66.7%)	0.088^a^
COVID-19 infection status of pregnant women and their relatives and friends	3(3.9%)	0(0.0%)	1.000^a^

Numbers in brackets refer to number of missing values

*BMI* body mass index, *COVID-19* 2019 coronavirus disease

^a^Fisher exact test

^b^Continuous correction of chi-square test

^c^The value are given as the number of participant or median with the percentage or interquartile range in parentheses, respectively

^d^Because these data were not normally distributed, the rank sum test was used for the comparison between groups

### Binary logistic regression analysis

As is shown in Tables 6, 7, 8 and 9, in the final analysis, the influence of current pregnancy status on movement (Mild vs. No, aORs were 3.89, *P* < 0.001, 2.92, *P* = 0.003, 1.58, *P* = 0.150 in the three trimesters, respectively; Severe vs. No, aORs were 13.00, 20.45, 5.38 in the three trimesters, respectively, all *P* < 0.05), and worries and fears about childbirth (aORs were 2.46, 2.96, 2.50 in the three trimesters, respectively, all *P* < 0.05) were associated with depression throughout the pregnancy. BMI (aOR = 2.13, *P* = 0.011) and history of physical diseases (aOR = 44.04, *P* = 0.023) were all positively associated with depressive symptoms independently during puerperium.

**Table 6:** Binary logistic regression analysis of risk factors for peripartum depression in the first trimester of pregnancy

Characteristics	aOR (95% CI)	***P***
Resident city	–	< 0.001
Beijing (Ref.)	–	–
Lanzhou	7.64(3.53-16.53)	< 0.001
Wuhan	2.35(0.74-7.43)	0.145
History of mental illness	43.20(2.97-628.77)	0.006
Vomiting during pregnancy	–	0.018
None (Ref.)	–	–
Mild (self-remission)	2.20(0.86-5.66)	0.102
Severe (ask for treatment)	5.93(1.72-20.43)	0.005
The influence of current pregnancy status on movement	–	< 0.001
No (Ref.)	–	–
Mild	3.89(1.94-7.81)	< 0.001
Severe	13.00(2.08-81.08)	0.006
Have worries and fears about childbirth	2.46(1.23-4.91)	0.011

*aOR* adjusted odds ratio, *CI* confidence interval

**Table 7:** Binary logistic regression analysis of risk factors for peripartum depression in the second trimester of pregnancy

Characteristics	aOR (95% CI)	***P***
Family income	–	0.003
80 thousand or below (Ref.)	–	–
80 thousand to 0.3 million	0.32(0.16-0.63)	0.001
More than 0.3 million	0.35(0.11-1.02)	0.054
The influence of current pregnancy status on movement	–	0.001
No (Ref.)	–	–
Mild	2.92(1.45-5.86)	0.003
Severe	20.45(3.10-135.01)	0.002
Have worries and fears about childbirth	2.96(1.58-5.54)	0.001
Requiring other people to help with daily tasks most of the time	0.34(0.17-0.66)	0.001
Living with parents-in-law	2.42 (1.14-5.14)	0.021

*aOR* adjusted odds ratio, *CI* confidence interval

**Table 8:** Binary logistic regression analysis of risk factors for peripartum depression in the third trimester of pregnancy

Characteristics	aOR (95% CI)	***P***
Resident city	–	< 0.001
Beijing (Ref.)	–	–
Lanzhou	3.24(1.44-7.31)	0.005
Wuhan	3.55(1.93-6.54)	< 0.001
Marital status (divorced/unmarried)	18.88(2.76-129.11)	0.003
Significant uterine contractions caused by anxiety	2.48(1.49-4.12)	< 0.001
The influence of current pregnancy status on movement	–	0.001
No (Ref.)	–	–
Mild	1.58(0.85-2.93)	0.150
Severe	5.38(2.21-13.08)	< 0.001
Have worries and fears about childbirth	2.50(1.51-4.14)	< 0.001
Requiring other people to help with daily tasks most of the time	0.55(0.31-0.97)	0.038

*aOR* adjusted odds ratio, *CI* confidence interval, *BMI* body mass index

**Table 9:** Binary logistic regression analysis of risk factors for peripartum depression in puerperal period

Characteristics	aOR (95% CI)	***P***
BMI	2.13 (1.19-3.82)	0.011
History of physical diseases	44.04(1.70-1141.17)	0.023

*aOR* adjusted odds ratio, *CI* confidence interval, *BMI* body mass index

## Discussion

To our best knowledge, this study is the first to explore peripartum depression and its related factors under the influence of the COVID-19 pandemic in China. The overall prevalence rate was 9.7%, with a relatively high prevalence in the first trimester (13.6%). From the perspective of gestational weeks, the most severe depressive symptoms occurred in the third week after delivery. In addition, independently related factors were different in the four stages of pregnancy/puerperium.

According to data reported before, up to 70% of women report symptoms of depression during pregnancy, and 10–16% fulfill criteria for peripartum depression [25]. In this study, the prevalence rate of peripartum depression was 9.7%, which was at the global average level, suggesting that during the period when confirmed COVID-19 cases were basically under control, pregnant women/puerperants may not have more depressive symptoms. Unfortunately, we did not have the same sample data to compare before the pandemic, so we could not draw this conclusion. This is just a speculation. The anti-COVID-19 pandemic is a long-term campaign. During the outbreak, the Chinese government has set up designated hospitals and conducted online consultation for this special group (pregnant women/puerperants) to reduce unnecessary outings. Therefore, their medical care needs, such as antenatal checkups, birth and postpartum examination were not affected. Other countries may refer to these measures, but the specific measures should be based on their own national conditions.

In addition, we found that the depressive symptoms during pregnancy were more common than those during puerperium, which was consistent with the results of previous studies [26, 27]. However, the specific prevalence rates were quite different. In this study, the prevalence rates of antepartum and postpartum depression were 9.8 and 7.3%, respectively. A review conducted by Gelaye et al. showed that in low-income and middle-income countries, they were 25.8 and 19.7%, respectively [27]. While in Sidebottom’s study, they were 15 and 6% among women served by urban community health centers, respectively [26]. This difference may be due to cultural differences, such as religious practices, nuclear or extended family structures [14]. Their surveys focused on Americans, while our data came from the three representative cities of Chinese mainland, which varied widely in culture, customs, and health care. In addition, different study designs may also cause this difference. Studies have shown that different assessment tools are associated with prevalence rates of antepartum depression [28]. Moreover, the prevalence rate of depression in the third trimester of pregnancy (7.9%) was much lower than that reported during the outbreak period (29.6%) [8]. This may be due to the fact that during our data collection period (February 28 to April 9, 2020), the COVID-19 in China was basically controlled, while Wu et al. conducted a survey on the prevalence of depression from January 1 to February 9, 2020 [8], when the pandemic was more serious. In addition, we had far more participants in the third trimester, compared to the other trimesters and postpartum. The reason may be that during the COVID-19 pandemic, most pregnant women chose not to go to the hospital as far as possible to reduce the risk of infection, while those who were about to give birth had to go to the hospital.

Besides, we also found that the prevalence rate of depression in the first trimester of pregnancy (13.6%) was higher than that in other stages, and the median PHQ-9 score in the 3 weeks after delivery was higher than that in other gestational weeks. However, a new systematic review shows that antepartum depression is the most common in the last trimester of pregnancy and the least in the second trimester [28]. This discrepancy may be caused by different participants and different screening tools. A total of 26 articles were included in the review, of which only one focused on the third trimester of pregnant woman in Taiwan (*n* = 153), and the rest (*n* = 28,095) were non-Chinese pregnant women. Different cultural differences may lead to differences in different rates. In addition, the most commonly used screening tool among the 26 articles included in the review is EPDS. Different screening tools may also cause differences in different rates. A study shows that there is a significant correlation between the gestational week and prevalence rate of depression [29]. This may also partly explain why we obtained different scores in different gestational periods or gestational weeks of pregnancy.

In addition to the two common independent risk factors during pregnancy (the influence of current pregnancy status on movement and worries and fears about childbirth), it should also be noted that the independent factors related to current depressive symptoms were different in the four stages. After all, each period has its own characteristics. In the first trimester of pregnancy, up to 80% of the pregnant women suffer from nausea and 50% of them suffer from vomiting or retching [30]. In the second trimester of pregnancy, the fetus develops more smoothly and grows faster relatively, and the mother’s overall symptoms are relatively stable [31]. In the third trimester of pregnancy, pregnant women are prone to fatigue and poor sleep quality [32]. During puerperium, hormone levels change greatly [33]. Different characteristics at different stages of pregnancy may lead to differences in women’s mood, hormone levels, life state, living environment, interpersonal relationship, etc., which may affect women’s depression symptoms, leading to different influencing factors at different stages of pregnancy. After all, when a life is conceived, it changes day by day. Every step of the way is not small.

Our study also has some limitations. First, this study is a cross-sectional design, which can only explore the relevant factors, but cannot draw causal conclusions. Second, the data collection time of this study is in a relatively stable stage of the domestic pandemic situation. In this way, the impact of COVID-19 on maternal depression may have been greatly weakened. Third, the sample size during puerperium is relatively small, which may lead us to fail to find the persuasive risk factors found in other studies. It is worth mentioning that the OR for history of physical diseases was much higher in the 4th stage of pregnancy, which may be due to the small sample size in stage 4 (*n* = 82). Besides, we got largest number of responders in the third trimester (*n* = 1042) when the depressive symptoms frequency was at its lowest level, which may not fully reflect the real prevalence rate of peripartum depression. Last but not least, the sampling method we used in this study was non-probability sampling, which is less valid than probability sampling. Therefore, our research sample may not be sufficiently representative. Considering these limitations of this study, in the next step, we will design a study using random sampling of national samples to further examine the correlation between these factors and peripartum depression, while strengthening the collection of postpartum women’s questionnaires to explore more convincing related factors as much as possible.

## Conclusions

In summary, our results revealed that the prevalence rate of peripartum depression was at a global average level under the influence of the COVID-19 pandemic, while the prevalence rate was the highest in the first trimester. This is an important supplement to the basic data of peripartum depression in Chinese women. Therefore, the public should not only pay attention to the postpartum depression in women, but also to depression in the early stage of pregnancy. Further, the independent factors related to peripartum depression were different at different stages of pregnancy. Thus, different interventions can be taken at different stages of pregnancy to alleviate the symptoms of peripartum depression.

## Data Availability

The datasets used and/or analyzed during the current study are available from the corresponding author on reasonable request.

## Abbreviations

PPD: Postpartum depression
COVID-19: 2019 coronavirus disease
QR: Quick response
PHQ-9: Patient Health Questionnaire-9
IQR: Interquartile range
OR: Odds ratio
CI: Confidence intervals
BMI: Body mass index
